# Floristic–Vegetational Features of *Geranium argenteum*, an Alpine–Apennine Species at Its Limit of Distribution in the Apennines

**DOI:** 10.3390/life13122273

**Published:** 2023-11-28

**Authors:** Sandro Ballelli, Giulio Tesei, Riccardo Pennesi, Marina Allegrezza

**Affiliations:** 1 Herbarium Universitatis Camerinensis, School of Biosciences and Veterinary Medicine, University of Camerino, Via Pontoni 5, 62032 Camerino, Italyriccardo.pennesi@unicam.it (R.P.); 2 Department of Agricultural, Food and Environmental Sciences, Marche Polytechnic University, Via Brecce Bianche, 60131 Ancona, Italy; m.allegrezza@univpm.it

**Keywords:** Alpine–Apennine range, biogeography, *Geranium argenteum*, syntaxonomy, vegetation

## Abstract

We present a floristic–vegetational study on a plant community dominated by *Geranium argenteum* in the Sibillini Mountains (Central Apennines), at the southern limit of its distribution in the Apennines. It is a rare pioneer community located at an elevation of about 2100 m a.s.l. with northern exposure on the fractured rocky ridges and at the edges of the rocky detrital valleys on lithosol, with a prolonged presence of snowpack and gelifraction processes. The results of the phytosociological analysis allow us to propose the new *Festuco italicae-Geranietum argentei* association referred to as the *Leontopodio nivalis-Elynion myosuroidis* alliance (*Carici rupestris-Kobresietea bellardii* class). The comparison with the Alpine and the Northern Apennines phytocoenoses characterized by *Geranium argenteum* allows us to provide a new interpretation of the syntaxonomical framework concerning the *Geranium argenteum* communities within its Alpine–Apennine range in light of the new data presented in this paper. The new *Festuco italicae-Geranietum argentei* association represents a further contribution to the knowledge of the relict alpine vegetation of the *Leontopodio nivalis-Elynion myosuroidis* alliance in the Sibillini Mountains and thus in the Central Apennines. Finally, habitat monitoring will be essential for assessing the impacts of climate change on this fragile and narrowly restricted plant community.

## 1. Introduction

**Figure 1:**
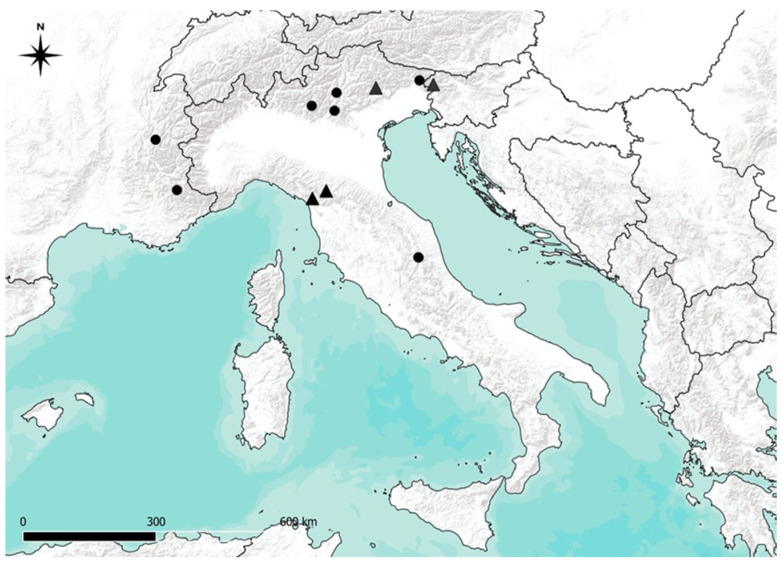
Geographical distribution map of *Geranium argenteum.* Circles (●) indicate the presence of *G. argenteum,* the triangles (▲) indicate the current position of the sites where the communities have been described.

*Geranium argenteum* is a rare Italian subendemic species distributed in the Alps (from Southeast France to Slovenia) [1] and within the Apennines ridges [2] (Figure 1). It is a small perennial herbaceous plant (8–15 cm) with all basal leaves arranged in a rosette and with a long and robust rhizome [2]. *Geranium argenteum* is a pioneer species that grows in open grasslands in neutral to alkaline conditions at nutrient-poor sites in high mountain environments [3,4], with the highest elevation in Val di Fassa (Dolomites Alps), where the species was found in a dolomitic scree at 2350 m a.s.l. [5]. The species occurs in ridge positions more or less exposed to the winds but also on scree slopes thanks to the long rhizome that can penetrate crevices and debris rock [6]. *Geranium argenteum* prefers calcareous substrate but is also present on siliceous lithologies in the Northern Apennines [7,8].

The origin and biogeographical history of this species are controversial, partly due to the absence of specific phylogenetic data in the literature. On the one hand, some authors indicate *G. argenteum* as a tertiary species (Gams 1933; Pignatti and Pignatti 2014; Chiarugi 1937) [5,9,10] with an Alpine–Mediterranean distribution, where the current discontinuity of geographic distribution should be considered a direct consequence of the glacial period that fragmented the primitive range of the species [5]. On the other hand, other authors indicate *G. argenteum* as a species of Alpine origin that migrated southward during the Quaternary glaciations (e.g., Ansaldi et al., 2008) [7].

In the Northern Apennines, the species has a scattered distribution in a few high-altitude sites [6,7,11], to the extent that it is listed as a protected species at the regional level [12,13]. Regarding the Central Apennines, lg. Ottaviani (in Bertoloni (1847): vol. VII pp. 224–225 [14]) reports the presence of *G. argenteum* “ex Umbria in montibus delle Fonti prope il Sasso Borghese” in the Sibillini Mountains group (Mt. Argentella). This record has not been confirmed in subsequent floristic studies; thus, for a long time, the Northern Apennines were indicated as the southernmost distribution limit of *G. argenteum* [2]. Only recently has the species been confirmed for the Central Apennines [15,16]; thus, this area automatically becomes the new southern distribution limit of *G. argenteum* in the Apennines.

Published literature data on plant communities with or dominated by *G. argenteum* mainly concern the Eastern Alpine chain (e.g., Surina 2005a; Sutter 1969) [17,18], while the published works on the Apennines exclusively concern the distribution and the size of populations at the regional level (e.g., Ansaldi et al., 2008) [7], except for a recent phytosociological study [19] on Mt. Cimone in the Northern Apennines (Figure 1). However, seven unpublished phytosociological relevés characterized by *G. argenteum* are reported in a thesis [11] for three Northern Apennines Mountain groups, one of which is from the Apuan Alps. Notably, a single relevé from the Apuan Alps (only one station, in the area of Pania della Croce) was also published in Ferrarini 1967 [6] (rel. n. 64 carried out on 14 September 1963).

From the syntaxonomic point of view, the phytosociological associations containing *G. argenteum* in their epithet currently recognized and sampled within the native range of the species are reported below with the original references at alliance and class levels: *Salici retusae-Geranietum argentei* Surina 2005 (*Soldanello-Salicion retusae* alliance, *Thlaspietea rotundifolii* class) for the Julian Alps in Slovenia [20,21]; *Sesleria sphaerocephala-Geranium argenteum* ass. prov. Sutter 1969 (*Oxytropido-Elynion* alliance; *Elyno-Seslerietea* class) (Sutter 1969), later reported as *Seslerio sphaerocephalae-Geranietum argentei* Sutter 1969 for the Dolomites [21,22] and as *Seslerio caeruleae-Geranietum argentei* Sutter 1969 prov for the Hautes Alpes in France [23]; and *Geranio argentei-Caricetum rupestris* Tomaselli, Foggi, Carbognani, Gennai, et Petraglia 2019 (*Oxytropido-Elynion* alliance; *Carici rupestris-Kobresietea bellardii* class) for the Northern Apennines [19].

The objectives of this research are: (i) the floristic–vegetational and ecological characterization of the communities dominated by *G. argenteum* in the Sibillini Mountain group (Central Apennines) at the southern limit of distribution of the species; (ii) to provide a new interpretation of the syntaxonomical framework concerning the *G. argenteum* communities within its Alpine–Apennine range in light of the new data presented in this paper.

## 2. Materials and Methods

### 2.1. Study Area

**Figure 2:**
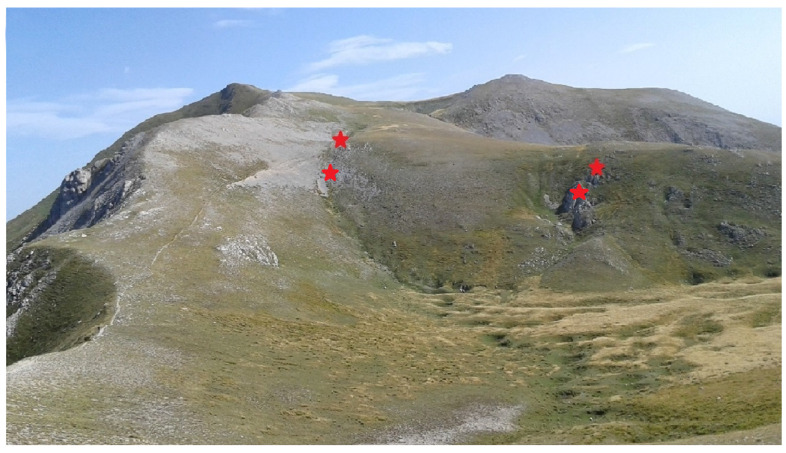
Plant landscape of the study area (the red stars indicate the locations of the relevés).

The study area is located on Mount Argentella in the Sibillini Mountain range and lies within the Natura 2000 Special Areas of Conservation (SAC IT5210071 “Monti Sibillini (versante umbro)”), included in the Monti Sibillini National Park. The substrate is exclusively calcareous, belonging to the Umbrian–Marche limestone succession and specifically to the Massive Limestone Formation [24]. The morphology is very rugged, with rocky bumps and ridges, scree, sinkhole systems, rocky detrital valleys, and snow beds (Figure 2).

The bioclimatic classification sensu Rivas-Martínez et al. (2011) [25] indicates a temperate macrobioclimate, oceanic bioclimate, and upper orotemperate thermotype [26]. The vegetation is represented by various types of high-altitude grassland communities depending on the morphology and stability of the substrate (Figure 2) and mainly referable to the following habitats of 92/43/EEC Directive Annex I: 6170 “alpine and subalpine calcareous grasslands” with plant communities belonging to the Apennine alliances *Leontopodio nivalis-Elynion myosuroidis*, *Seslerion apenninae*, and *Ranunculo pollinensis-Nardion strictae*; 8120 “Calcareous and calcshist screes of the montane to alpine levels (*Thlaspietea rotundifolii*)” with plant communities belonging to the Apennine alliance *Linario-Festucion dimorphae*; and 8210 “Calcareous rocky slopes with chasmophytic vegetation” with plant communities belonging to the Apennine alliance *Saxifragion australis* (*Asplenietea* class).

### 2.2. Vegetation Study

The study of the vegetation was conducted according to the Braun-Blanquet phytosociological method [27]. Each vascular plant detected in the survey area was associated with an abundance value using the seven-point Braun-Blanquet scale according to the following cover values: with cover <1% (r, very few individuals; +, few individuals) and with cover ≥1% (1: from 1% to 5%; 2: from 5% to 25%; 3: from 25% to 50%; 4: from 50% to 75%; 5: from 75% to 100%). The nomenclature of the species follows Bartolucci et al. (2018) [13] with the exception of *Luzula italica*, which follows Pignatti et al. (2017–2019) [2]. The phytosociological nomenclature follows the rules of the ICPN [28]. The chorological types refer to Pignatti et al. (2017–2019) [1] and Aeschimann et al. (2004) [2]. The chorological types were grouped as follows: endemic (endemic Italian; endemic Alps; subendemic), Mediterranean (Eurimediterranean; Mediterranean–mountain; amphi-Adriatic), Eurasian (Eurasian; European (center, south, southeast); European Caucasian; paleotemperate; southwest Asiatic–Mediterranean), center-European orophytes (European orophytes (center, west); Eurasian orophytes; east Alpine–Carpatian), south-European orophytes (European orophytes (south, southeast, southwest); Alpine–Pyrenaic), boreal (Eurosiberian; circumboreal; Arctic–Alpine), and cosmopolitan (cosmopolitan; subcosmopolitan).

A total of 106 phytosociological relevés were used for the comparisons with the literature data. Four of these were unpublished and were performed in the study area.

The syntaxonomic nomenclature follows Biondi et al. (2014, 2015) [29,30] for the *Festuco-Seslerietea* class, Chytrý et al. (2015) [31] for the *Carici rupestris-Kobresietea bellardii* class, and Mucina et al. (2016) [32] the for the other classes.

The plant collections of the new association are preserved at Herbarium Universitatis Camerinensis, School of Biosciences and Veterinary Medicine, University of Camerino, Via Pontoni 5, 62032 Camerino, Italy.

### 2.3. Statical Analysis

Braun-Blanquet’s cover-abundance values were transformed according to the van der Maarel scale [33] and then subjected to multivariate analysis through the use of the VEGAN community ecology package [34] for R software version 4.2.3 [35]. In order to assess the degree of similarity between the surveys carried out in the study area and those already published on plant communities in the alpine and subalpine planes of the Central Apennines, a cluster analysis was carried out by applying Ward’s minimum variance method to the stratified similarity matrix calculated using the Bray–Curtis index. To analyze the changes in species composition between the different *G. argenteum* communities, principal component analysis (PCA) was performed. Furthermore, the “indicspecies” R package [36] was used to perform the indicator species analysis [37], in order to identify the species significantly associated with the different *G. argenteum* communities.

## 3. Results and Discussion

### 3.1. Geranium argenteum Community in the Study Area

Association: *Festuco italicae-Geranietum argentei ass. nov*. (*typus* rel. n. 2 of Table 1)

Dominant species: *Geranium argenteum*

Constant species: *Armeria gracilis* subsp. *gracilis*, *Carex kitaibeliana*, *Festuca violacea* subsp. *italica*, *Gentianella columnae*, *Geranium argenteum*, *Poa molinerii*, *Plantago atrata*, *Potentilla crantzii*, *Ranunculus breyninus*, *Sabulina verna* subsp. *verna*, *Saxifraga adscendens* subsp. *adscendens*, *Sedum atratum*, *Silene acaulis* subsp. *bryoides*.

Diagnostic species: *Geranium argenteum* (characteristic species), *Gentianella columnae* (differential species), *Potentilla brauneana*, (differential species), *Festuca violacea* subsp. *italica* (differential species), and *Achillea barrelieri* (transgressive species).

**Figure 3:**
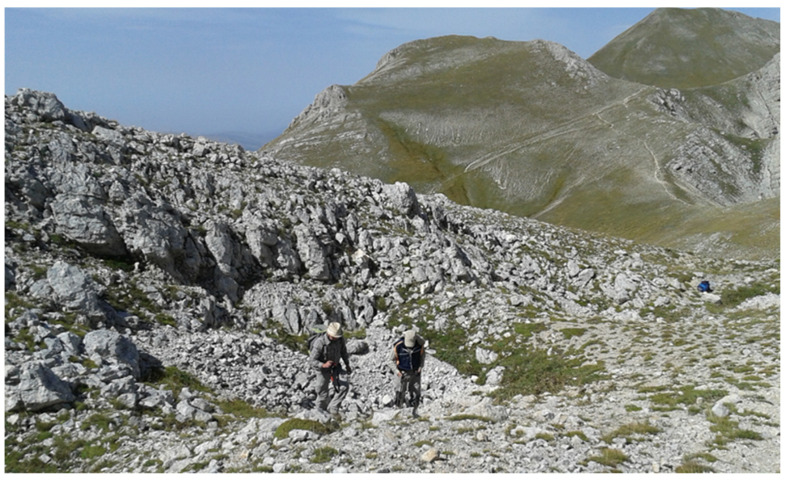
The typical habitat of the *G. argenteum* in the study area.

**Figure 4:**
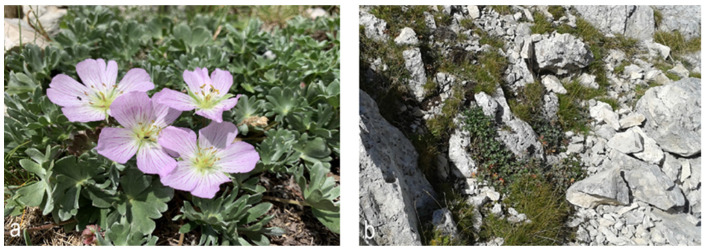
*G. argenteum* close-up of flowering (**a**) and *G. argenteum* community in the study area on the fractured rocky ridges (**b**).

It is an extremely rare and fragmentary pioneer community dominated by *G. argenteum* (mean cover 44%) with *Festuca violacea* subsp. *italica* and *Silene acaulis* subsp. *bryoides* that develops exclusively near the summit sector of Monte Argentella (2201 m a.s.l.) in the northern aspects and at an elevation between 2040 and 2080 m a.s.l. (Figure 2 and Figure 3). It is present on the fractured rocky ridges (Figure 4) and at the edges of the rocky detrital valleys on lithosol, in conditions of prolonged presence of the snowpack and gelifraction processes. Thanks to its long and robust rhizome, *G. argenteum* creeps into the cracks of the pebbles up to the consolidated soil also associated with *Silene acaulis* subsp. *bryoides*.

The elaboration of the phytosociological relevés and the comparison with the plant communities occurring in the alpine and subalpine belts of the Central Apennines, which exhibit floristic similarities with the *G. argenteum* community in the study area (Table S1), highlight the floristic–vegetational autonomy of the *G. argenteum* community under investigation, for which the new *Festuco italicae-Geranietum argentei* association is proposed (Table 1).

**Table 1:** *Festuco italicae*-*Geranietum argentei ass. nov.* (*typus* rel. n° 2, indicated by asterisk).

No. of Relevés	1	2 *	3	4	
Elevation (m a.s.l.)	2080	2076	2036	2040	
Aspect	N	N	N	N	p
Slope (°)	40	35	30	35	r
Area (mq)	40	10	50	15	e
Cover total %	70	70	70	30	s.
No. of Species	40	31	44	28	
***Festuco italicae-Geranietum argentei***					
*Geranium argenteum* L.	3	4	4	2	4
*Festuca violacea* Ser. *ex* Gaudin subsp. *italica* Foggi, Gr. Rossi *et* Signorini	3	2	2	1	4
*Gentianella columnae* (Ten.) Holub	+	1	1	+	4
*Achillea barrelieri* (Ten.) Sch. Bip. subsp. barrelieri	+	+	+	.	3
*Potentilla brauneana* Hoppe	.	+	+	+	3
***Leontopodio-Elynion*, *Oxytropido-Elynetalia*, *Carici rupestris-Kobresietea bellardii***					
*Silene acaulis* (L.) Jacq. subsp. *bryoides* (Jord.) Nyman	+	2	2	1	4
*Carex kitaibeliana* Degen *ex* Bech.	1	1	1	+	4
*Sabulina verna* (L.) Rchb. subsp. *verna*	+	+	+	+	4
*Potentilla crantzii* (Crantz) Beck *ex* Fritsch subsp. *crantzii*	+	+	+	+	4
*Sedum atratum* L.	+	+	+	+	4
*Erigeron epiroticus* (Vierh.) Halácsy	+	+	+	.	3
*Leontopodium nivale* (Ten.) Hand.-Mazz.	+	+	.	.	2
*Omalotheca diminuta* (Braun-Blanq.) Bartolucci *et* Galasso	.	+	.	+	2
***Festuco-Seslerietea***					
*Ranunculus breyninus* Crantz	+	+	+	+	4
*Euphrasia salisburgensis* Funck *ex* Hoppe	+	+	1	.	3
*Edraianthus graminifolius* (L.) A. DC. subsp. *graminifolius*	+	+	+	.	3
*Bellidiastrum michelii* Cass.	1	+	1	.	3
*Draba aizoides* L. subsp. *aizoides*	+	.	+	+	3
*Pulsatilla alpina* (L.) Delarbre subsp. *millefoliata* (Bertol.) D.M. Moser	+	.	+	.	2
*Sesleria juncifolia* Wulfen *ex* Suffren subsp. *juncifolia*	+	.	+	.	2
*Androsace villosa* L. subsp. *villosa*	.	+	+	.	2
*Alchemilla nitida* Buser	.	.	+	+	2
*Gentiana verna* L. subsp. *verna*	.	.	+	+	2
*Aster alpinus* L. subsp. *alpinus*	+	.	.	.	1
*Carduus defloratus* L. subsp. *carlinifolius* (Lam.) Ces.	+	.	.	.	1
*Pedicularis elegans* Ten.	+	.	.	.	1
*Paronychia kapela* (Hacq.) A. Kern. subsp. *kapela*	+	.	.	.	1
*Trinia dalechampii* (Ten.) Janch.	.	+	.	.	1
***Nardetea strictae* and *Juncetea trifidii***					
*Plantago atrata* Hoppe subsp. *atrata*	+	+	+	+	4
*Taraxacum apenninum* (Ten.) DC.	.	+	+	+	4
*Crepis aurea* (L.) Cass. subsp. *glabrescens* (Caruel) Arcang.	.	+	1	+	4
*Pilosella lactucella* (Wallr.) P.D. Sell *et* C. West subsp. *nana* (Scheele) M. Laínz	.	.	+	.	1
*Luzula italica* Parl.	.	.	.	+	1
*Botrychium lunaria* (L.) Sw.	.	.	.	+	1
***Asplenietea trichomanis***					
*Cystopteris fragilis* (L.) Bernh.	+	.	+	+	2
*Saxifraga paniculata* Mill.	+	.	+	+	2
*Campanula tanfanii* Podlech	+	.	+	.	2
*Asplenium viride* Huds.	.	.	+	+	2
***Thlaspietea rotundifolii***					
*Saxifraga adscendens* L. subsp. adscendens	+	+	+	+	4
*Doronicum columnae* Ten.	+	.	+	+	2
*Galium magellense* Ten.	+	.	.	.	1
*Robertia taraxacoides* (Loisel.) DC.	+	.	.	.	1
*Ranunculus brevifolius* Ten.	+	.	.	.	1
*Leucopoa dimorpha* (Guss.) H. Scholz *et* Foggi	.	.	+	.	1
*Arabis alpina* L. subsp. *caucasica* (Willd.) Briq.	.	.	.	+	1
**Other species**					
*Armeria gracilis* Ten. subsp. *gracilis*	1	1	+	+	4
*Poa molinerii* Balb.	1	+	+	1	4
*Globularia meridionalis* (Podp.) O. Schwarz	+	+	+	.	3
*Helictochloa praetutiana* (Parl. *ex* Arcang.) Bartolucci, F. Conti, Peruzzi *et* Banfi subsp. *praetutiana*	+	+	+	.	3
*Koeleria australis* A. Kern	+	+	+	.	3
*Thymus praecox* Opiz subsp. *polytrichus* (A. Kern *ex* Borbás) Jalas	1	+	1	.	3
*Oreojuncus monanthos* (Jacq.) Záv. Drábk. *et* Kirschner	+	.	+	.	2
*Anthyllis vulneraria* L. subsp. *nana* (Ten.) Tammaro	.	+	+	.	2
*Cerastium arvense* L. subsp. *suffruticosum* (L.) Ces.	.	.	+	+	2
*Ziziphora granatensis* (Boiss. et Reut.) Melnikov subsp. *alpina* (L.) Bräuchler et Gutermann	1	.	.	.	1
*Helianthemum oelandicum* (L.) Dum. Cours. subsp. *incanum* (Willk.) G. López	+	.	.	.	1
Myosotis graui Selvi	.	+	.	.	1
*Cynanchica pyrenaica* (L.) P. Caputo *et* Del Guacchio subsp. *neglecta* (Guss.) P. Caputo *et* Del Guacchio	.	.	+	.	1
*Carlina acaulis* subsp. *caulescens* (Lam.) Schübl. *et* G. Martens	.	.	+	.	1
*Anthyllis montana* L. subsp. *jacquinii* (Rchb. *f*.) Rohlena	.	.	+	.	1

Dates, localities, and geographical coordinates (WGS84–UTM 33T) of relevès performed in the study area: rel. 1, Mt. Argentella, 356137 m E 4747032 m N; rel. 2, Mt. Argentella, 356136 m E 4747040 m N; rel. 3, Mt. Argentella, 355935 m E 4747127 m N; rel. 4, Mt. Argentella, 355931 m E 4747114 m N.

The specific combination that is characteristic of the new association includes: *Geranium argenteum*, *Festuca violacea* subsp. *italica*, *Gentianella columnae*, *Achillea barrelieri*, and *Potentilla brauneana*. *Geranium argenteum* is an Alpine–Apennine subendemic species at its southern limit of distribution in the Apennines, and *F. violacea* subsp. *italica*, *G. columnae*, and *A. barrelieri* subsp. *barrelieri* are endemic Italian species restricted to the Central Apennines. Finally, *P. brauneana* is an Alpine–Pyrenaic species first reported in the Sibillini Mountains and new to the Umbrian flora; this species is very rare in the Central Apennines, reaching the southern limit of its distribution in the Mainarde Mountain group (Molise Region) [38]. All these species testify to the high biogeographic value of the new association, which has a strong component of Italian endemic species and subendemic species (25%) (Table S2). The boreal species including Arctic–Alpine species, such as *Potentilla crantzii* subsp. *crantzii*, are well represented (15%). Also important is the role of the south-European orophytes (38%), including Southeast European orophytes species exhibiting a strict amphi-Adriatic distribution, such as *Carex kitaibeliana*. The Mediterranean species such as *Sesleria juncifolia* are represented well in terms of richness and frequency (11.7% and 9.8%, respectively) but negligible in cover percentage (1.8%) (Table S2).

The classification of the association at a higher syntaxonomic level is quite problematic due to the ecology nature of *G. argenteum* itself, which is intermediate between four vegetation classes (*Carici rupestris-Kobresietea bellardii*, *Festuco-Seslerietea*, *Juncetea trifidi*, and *Thlaspietea rotundifolii*) [17,18,19]. Among these, in the area under investigation, the most represented classes are the *Carici rupestris-Kobresietea bellardii* and *Festuco-Seslerietea* (Table 1). Based on the classification of the relevé groups of the plant communities in the central Apennines (Figure S1), referred to as the *Festuco-Seslerietea* (*Seslerion apenninae* and *Carici humilis-Seslerion apenninae* alliances) (Figure S1, cluster I) and *Carici rupestris-Kobresietea bellardii* (*Leontopodio nivalis-Elynion myosuroidis* alliance) classes (Figure S1, cluster II), the new association *Festuco italicae-Geranietum argentei* has been referred to the *Leontopodio nivalis-Elynion myosuroidis* alliance of the *Carici rupestris-Kobresietea bellardii* class. Among the species identified as characteristic of this alliance are *Silene acaulis* subsp. *bryoides*, *Achillea barrelieri* subsp. *barrelieri*, *Erigeron epiroticus*, *Leontopodium nivale*, and *Omalotheca diminuta* (the latter reported in Blasi 2003 and Lancioni et al., 2011 [39,40]). *Carex kitaibeliana* instead assumes the significance of differential species of the *Leontopodio nivalis-Elynion myosuroidis* alliance compared to *Oxytropido-Elynion* (Table 1 and Table S1). The species that primarily belong to the class *Festuco-Seslerietea* highlight the contact of the *G. argenteum* community with the grasslands of the order *Seslerietalia tenuifoliae*.

The results of the classification are in accordance with the list of the syntaxa of the Central Apennines reported in the *Leontopodio nivalis-Elynion myosuroidis* alliance [31], with the exception of the *Seslerio apenninae-Dryadetum octopetalae* association, which in this classification (Figure S1) is included in the floristic context of the *Seslerion apenninae* alliance, confirming the original interpretation of the authors [41] and the subsequent revision of Lancioni et al. (2011) [40].

The plant communities included in the *Leontopodio nivalis-Elynion myosuroidis* alliance (Table S1) may be interpreted as relict communities of the primary alpine tundra on the summits of the highest mountains in the Central Apennines [31], which show an evident alpine belt, such as Gran Sasso [41,42,43] and Majella [39,44,45], while in the Sibillini Mountains, as in other mountain groups in the Central Apennines where the alpine belt is reduced, they appear to be very fragmentary [46,47]. The new *Festuco italicae-Geranietum agentei* association referred to the *Leontopodio nivalis-Elynion myosuroidis* represents a further contribution to the knowledge of the alpine vegetation in the Sibillini Mountains and thus in the Central Apennines.

The new plant association refers to the following habitat of community interest (Directive 92/43/EEC Annex I): 6170 “Alpine and subalpine calcareous grasslands” sub-type 36.42 “Wind edge naked-rush swards” (CORINE Biotopes) (corresponding to R45 EUNIS 2019/2021 code).

### 3.2. Comparison between the Geranium argenteum Communities in the Alpine–Apennine Biogeographical Range

In order to provide a new interpretation of the syntaxonomic framework concerning the communities of *G. argenteum* within its Alpine–Apennine range and to identify their ecological and floristic characteristics, the new association *Festuco italicae-Geranietum argentei* from the study area was compared with *Salici retusae-Geranietum argentei* from the Julian Alps in northwestern Slovenia, *Sesleriello sphaerocephalae-Geranietum argentei* from the Dolomites Alps, *Geranio argentei-Caricetum rupestris* from the Tuscan-Emilian Apennines, and the *Geranium argenteum* group from the Northern Apennines (six relevés, from the Tuscan-Emilian Apennines on siliciclastic substrate, and one from the Apuan Alps (Pania della Croce) on calcareous substrate).

#### 3.2.1. Topography and *Geranium argenteum* Cover

The Alpine–Apennine communities characterized by *G. argenteum* are present on snow-covered scree slopes, snow valleys, and windy rocky ridges in an elevation range from 1450 to 2130 m a.s.l., with the highest average values (always above 2000 m a.s.l.) in the pioneer communities of *Sesleriello sphaerocephalae-Geranietum argentei* (original name: *Sesleria sphaerocephala-Geranium argenteum* Sutter 1969 *ass. prov*. (*nom. inv.* Art. 1)) in the Dolomites and *Festuco italicae-Geranietum argentei* in the Central Apennines (Table 2). The substrate is calcareous, except for the *Geranio argentei-Caricetum rupestris* association and *Geranium argenteum* group in the Northern Apennines (Tuscan-Emilian sector), which are siliciclastic (Table 2). It should be noted that the *G. argenteum* group includes six relevés from the Tuscan-Emilian Apennines on a siliciclastic substrate and one relevé from the Apuan Alps on calcareous lithologies. On average, the total cover of the species is 75% (the highest in *Sesleriello sphaerocephalae-Geranietum argentei*), while the average number of species is 26 (the highest in the *G. argenteum* group). The coverage of *G. argenteum* (Table 2) reaches significantly high average values in the communities of *Festuco italicae-Geranietum argentei* followed by that of *Salici retusae-Geranietum argentei*, while the lowest value was recorded in *Geranium argentei-Caricetum rupestris*.

#### 3.2.2. Floristic–Vegetational Features with Syntaxonomical Notes

**Figure 5:**
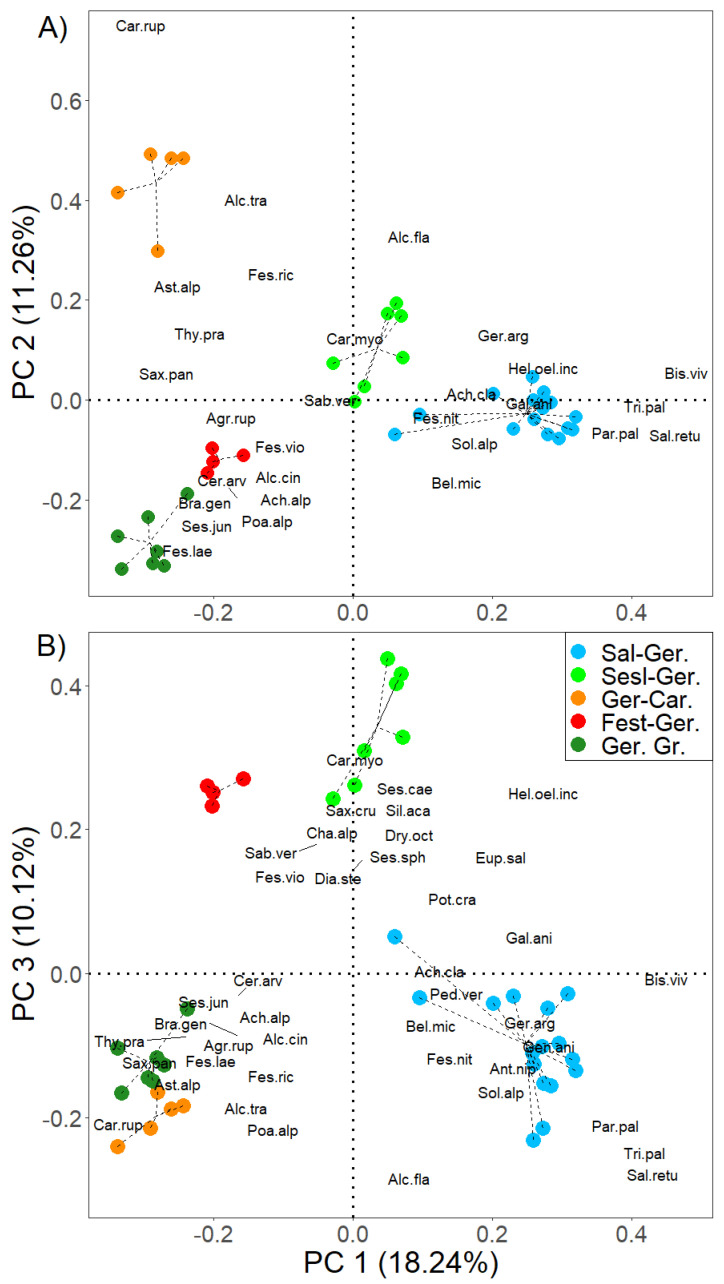
PCA of the relevés belonging to the 5 *Geranium argenteum* communities (total variance explains 72.2%; eigenvalue and variance explained by the first three axes: PC1 0.13 and 18.2%; PC2 0.08 and 11.2%; PC3 0.07 and 10.1%); only the species significantly linked to the 5 communities derived from the indicator species analysis (Table 3) with score >0.15 are shown. Axes 1–2 (**A**) and axes 1–3 (**B**). Information on the weight of each species with respect to the main components identified, as well as the percentage of variance explained by each of them, is shown in Table S3. Abbreviations: Sal-Ger. (*Salici retusae-Geranietum argentei*); Sesl-Ger. (*Sesleriello sphaerocephalae-Geranieum argentei*); Ger-Car. (*Geranio argentei-Caricetum rupestris*); Fest-Ger. (*Festuco italicae-Geranietum argentei*, study area); Ger. gr. (*Geranium argenteum* group). The full names of the abbreviated species in the figure are reported in Table 3.

The PCA of the groups of relevés, belonging to the five Alpine–Apennine communities characterized by *G. argenteum* (Figure 5), underlines the floristic, biogeographic, and syntaxonomic context of the plant communities even more clearly.

*Salix retusa* and *Bistorta vivipara* guide the floristic variation along the main PC1 axis (Figure 5A) and characterize the alpine cenoses on calcareous substrates of *Salici retusae-Geranietum argentei*, differentiated by boreal species and European and south-European orophytes of the *Soldanello minimae-Salicion retusae* alliance (*Arabidetalia coeruleae* order), whose association is originally referred to by the authors [17].

*Carex rupestris* guides the floristic variation along the PC2 axis, with the clear separation of the cenosis of the windy summit ridges of the Northern Apennines (Tuscan-Emilian sector) on a siliciclastic substrate of *Geranio argentei-Caricetum rupestris*, characterized by the high coverage of *Carex rupestris* of the *Carici rupestris-Kobresietea bellardii* class and silicicolous species of the *Juncetea trifidii* class, including *Festuca riccierii*, a species endemic to the Northern Apennines [19].

Along the third axis (PC3) of the PCA (Figure 5B), *Carex myosuroides* guides the floristic variation. There is an evident separation of the Alpine and Apennine pioneer cenoses on the calcareous substrate of the *Sesleriello sphaerocephalae-Geranieum argentei* and *Festuco italicae-Geranietum argentei* associations characterized by species of the *Carici rupestris-Kobresietea bellardii* class and *Festuco-Seslerietea* class from those on the siliciclastic substrate. In particular, numerous Arctic–Alpine species, typical of the *Carici rupestris-Kobresietea bellardii* class and *Oxytropido-Elynion* alliance, are significantly linked to the communities of the alpine windy ridges of *Sesleriello sphaerocephalae-Geranietum argentei* (Table 3), such as *Carex myosuroides*, *S. acaulis*, *Dryas octopetala*, *Chamorchis alpina*, and *Gentiana nivalis.* This may confirm the syntaxonomic classification of the association in the *Oxytropido-Elynion* alliance (*Carici rupestris-Kobresietea bellardii* class) as reported by Sutter (1969) [18].

In addition to the boreal species of the *Carici-Kobresietea* class, such as *P. crantzii* subsp. *crantzii* and *Sedum atratum*, the Italian endemic (*Achillea barrelieri*) and subendemic species (*Leontopodium nivale*), Mediterranean elements (*Erigeron epiroticus*), and south-European orophytes species (*Omalotheca diminuta*, *Carex kitaibeliana*) that do not reach the Alps are significantly linked to the pioneer calcicolous communities of *Festuco italicae-Geranietum argentei* (Table 1 and Table 3) in the Central Apennines that, in turn, are also characteristics and differentials of the *Leontopodio nivalis-Elynion myosuroidis* Central Apennines alliance to which the association refers.

Regarding the *G. argenteum* group communities of the Northern Apennines (Tuscan-Emilian Apennines and Apuan Alps sectors), ranging from the upper supratemperate to the lower orotemperate thermotype, there are significant species (Table 3) of the *Festuco-Seslerietea* (e.g., *Sesleria juncifolia* complex) and *Festuco-Brometea* classes (e.g., *Cerastium arvense* subsp. *suffruticosum*). To these, exclusively on siliciclastic substrate (Tuscan-Emilian sector), the species of *Nardetea strictae* classes are added (e.g., *Antennaria dioica*), and those of the *Juncetea trifidi* classes (e.g., *Agrostis rupestris*) and *Loiseleurio-Vaccinietea* classes (e.g., *Vaccinium uliginosum*), which indicate the connection with the cenoses of *Geranium argentei-Caricetum rupestris* of the highest elevation. An exception is the only relevé from the Apuan Alps [11], on a calcareous substrate in which the acidophilic species typical of siliciclastic rocks of the *Nardetea strictae*, *Juncetea trifidi*, and *Louseleurio-Vaccinetea* classes are practically absent.

**Table 3:** Species significantly linked to the plant communities characterized by *Geranium argenteum* deriving from the indicator species analysis (*p* value < 0.05, significance codes: *** ≤ 0.001, ** ≤ 0.01, * ≤ 0.05; column A is the positive predictive value of the species as indicator of the site group; column B is the sensitivity of the species as indicator of the target site group). The diagnostic species indicated in the original description of the association are underlined.

Plant Association	Abbrev.	A	B	Stat.	*p* Value		* Syntaxonomical Reference
**Salici retusae-Geranietum argentei (Eastern Alps; Julian Alps)**							
*Salix retusa L.*	Sal.retu	1.000	0.813	0.901	0.001	***	*Thlaspietea rotundifolii*
*Trifolium pallescens * Schreb.	Tri.pal	1.000	0.875	0.935	0.001	***	*Thlaspietea rotundifolii*
*Soldanella alpina* L.	Sol.alp	0.747	0.500	0.611	0.028	*	*Thlaspietea rotundifolii*
*Bistorta vivipara * (L.) Delarbre	Bis.viv	0.603	0.875	0.726	0.001	***	*Carici rupestris-Kobresietea bellardii*
*Gentianella anisodonta* (Borbás) Á. Löve *et* D. Löve	Gen.ani	1.000	0.688	0.829	0.001	***	*Festuco-Seslerietea*
*Geranium argenteum* L.	Ger.arg	0.306	1.000	0.553	0.001	***	*Festuco-Seslerietea*
*Erigeron glabratus* Hoppe *et* Hornsch. *ex* Bluff *et* Fingerh.	Eri.gla	1.000	0.438	0.661	0.013	*	*Festuco-Seslerietea*
*Festuca nitida* Kit. *ex* Schult. subsp. *nitida*	Fes.nit	1.000	0.438	0.661	0.015	*	*Festuco-Seslerietea*
*Galium anisophyllon* Vill.	Gal.ani	0.549	0.625	0.586	0.043	*	*Festuco-Seslerietea*
*Anthoxanthum nipponicum* Honda	Ant.nip	1.000	0.500	0.707	0.009	**	*Juncetea trifidii*
*Parnassia palustris * L.	Par.pal	1.000	0.875	0.935	0.001	***	Other
**Sesleriello sphaerocephalae-Geranietum argentei (Eastern Alps; Dolomites)**							
*Carex capillaris * L.	Car.cap.	0.727	0.428	0.558	0.047	*	*Carici rupestris-Kobresietea bellardii*
*Dryas octopetala* L. subsp. *octopetala*	Dry.oct	0.926	0.714	0.813	0.001	***	*Carici rupestris-Kobresietea bellardii*
*Silene acaulis* (L.) Jacq. (*s.l.*)	Sil.aca	0.901	0.857	0.879	0.001	***	*Carici rupestris-Kobresietea bellardii*
*Carex myosuroides * Vill. (*Elyna myosuroides* (Vill.) Fritsch)	Car.myo	1.000	0.714	0.845	0.002	**	*Carici rupestris-Kobresietea bellardii*
*Chamorchis alpina* (L.) Rich.	Cha.alp	1.000	0.714	0.845	0.002	**	*Carici rupestris-Kobresietea bellardii*
*Festuca pumila* Chaix	Fes.pum	1.000	0.429	0.655	0.020	*	*Carici rupestris-Kobresietea bellardii*
*Gentiana nivalis* L.	Gen.niv	0.821	0.429	0.593	0.033	*	*Carici rupestris-Kobresietea bellardii*
*Helianthemum oelandicum* (L.) Dum. Cours. subsp. *alpestre* (Jacq.) Ces.	Hel.oel.alp	0.677	1.000	0.823	0.001	***	*Festuco-Seslerietea*
*Anthyllis vulneraria* L. subsp. *alpicola* (Brügger) Gutermann	Ant.vul.alp	1.000	0.571	0.756	0.001	***	*Festuco-Seslerietea*
*Dianthus sternbergii * Sieber ex Capelli	Dia.ste	1.000	0.571	0.756	0.004	**	*Festuco-Seslerietea*
*Sesleria caerulea* (L.) Ard.	Ses.cae	1.000	0.857	0.926	0.002	**	*Festuco-Seslerietea*
*Sesleriella sphaerocephala * (Ard.) Deyl (*Sesleria sphaerocephala* Ard.)	Ses.sph	1.000	0.571	0.756	0.003	**	*Festuco-Seslerietea*
*Selaginella selaginoides* (L.) P. Beauv. *ex* Schrank *et* Mart.	Sel.sel	0.746	0.571	0.653	0.023	*	*Festuco-Seslerietea*
*Carex firma* Host	Car.fir	0.889	0.429	0.617	0.035	*	*Festuco-Seslerietea*
*Cherleria sedoides* L.	Che.sed	0.774	0.429	0.576	0.044	*	*Juncetea trifidii*
*Saxifraga crustata* Vest.	Sax.cru	1.000	0.714	0.845	0.001	***	*Asplenietea trichomanis*
*Oxytropis carinthica * Fischer	Oxy.car	1.000	0.429	0.655	0.017	*	Other
*Polygala chamaebuxus* L.	Pol.cha	1.000	0.429	0.655	0.027	*	Other
**Geranio argentei-Caricetum rupetris (Northern Apennines)**							
*Carex rupestris* All.	Car.rup	0.901	1.000	0.949	0.001	***	*Carici rupestris-Kobresietea bellardii*
*Aster alpinus * L. subsp. *alpinus*	Ast.alp	0.549	0.800	0.663	0.014	*	*Festuco-Seslerietea*
*Alchemilla transiens* (Buser) Buser	Alc.tra	1.000	0.800	0.894	0.001	***	*Juncetea trifidii*
*Festuca riccerii* Foggi et Gr. Rossi	Fes.ric	1.000	0.600	0.775	0.002	**	*Juncetea trifidii*
*Luzula lutea* (All.) DC. subsp. *lutea*	Luz.lut	0.808	0.600	0.696	0.004	**	*Juncetea trifidii*
*Alchemilla flabellata* Buser	Alc.fla	0.535	0.800	0.654	0.022	*	*Juncetea trifidii*
**Geranium argenteum group (Northern Apennines)**							
*Campanula cochleariifolia* Lam.	Cam.coc	1.000	0.571	0.756	0.002	**	*Thlaspietea rotundifolii*
*Anemonastrum narcissiflorum* (L.) Holub	Ane.nar	0.920	0.714	0.810	0.002	**	*Festuco-Seslerietea*
*Sesleria juncifolia* Wulfen *ex* Suffren subsp. *juncifolia*	Ses.jun	0.767	0.714	0.740	0.002	**	*Festuco-Seslerietea*
*Alchemilla cinerea* Buser	Alc.cin	1.000	0.857	0.926	0.001	***	*Festuco-Seslerietea*
*Achemilla alpina* L. Series *saxatiles* Buser	Ach.alp	1.000	0.857	0.926	0.001	***	*Juncetea trifidii*
*Oreojuncus trifidus* (Jacq.) Záv. Drábk. *et* Kirschner	Ore.tri	1.000	0.429	0.655	0.027	*	*Juncetea trifidii*
*Pedicularis tuberosa* L.	Ped.tub	1.000	0.429	0.655	0.025	*	*Juncetea trifidii*
*Phyteuma hemisphaericum* L.	Phy.hem	0.811	0.429	0.589	0.040	*	*Juncetea trifidii*
*Agrostis rupestris* All.	Agr.rup	0.616	0.571	0.594	0.028	*	*Juncetea trifidii*
*Viola cavillieri* W. Becker	Vio.cav	1.000	0.429	0.655	0.013	*	*Juncetea trifidii*
*Gentianella campestris* (L.) Börner	Gen.cam	1.000	0.429	0.655	0.018	*	*Loiseleurio-Vaccinietea*
*Juniperus communis* L. subsp. *nana* (Willd.) Syme	Jun.com.nan	1.000	0.429	0.655	0.025	*	*Loiseleurio-Vaccinietea*
*Vaccinium uliginosum* L. subsp. *microphyllum* (Lange) Tolm.	Vac.uli	0.821	0.429	0.593	0.035	*	*Loiseleurio-Vaccinietea*
*Antennaria dioica* (L.) Gaertn.	Ant.dio	1.000	0.571	0.756	0.004	**	*Nardetea strictae*
*Avenella flexuosa* (L.) Parl. subsp. *flexuosa*	Ave.fle	1.000	0.571	0.756	0.002	**	*Nardetea strictae*
*Cynanchica aristata* (L. *f*.) P. Caputo *et* Del Guacchio (*s.l*.)	Asp.ari	1.000	0.714	0.845	0.001	***	*Festuco-Brometea*
*Brachypodium genuense* (DC.) Roem. *et* Schult.	Bra.gen	1.000	0.857	0.926	0.001	***	*Festuco-Brometea*
*Festuca laevigata* Gaudin subsp. *laevigata*	Fes.lae	1.000	0.857	0.926	0.001	***	*Festuco-Brometea*
*Cerastium arvense* L. subsp. *suffruticosum* (L.) Ces.	Cer.arv	0.667	0.857	0.756	0.002	**	*Festuco-Brometea*
*Euphrasia stricta* D. Wolff *ex* J.F. Lehm.	Eup.str	1.000	0.571	0.756	0.003	**	*Festuco-Brometea*
*Pilosella officinarum* Vaill.	Hie.pil	1.000	0.571	0.756	0.002	**	*Festuco-Brometea*
*Poa alpina* L. subsp. *alpina*	Poa.alp	0.692	1.000	0.832	0.001	***	Other
*Luzula campestris* (L.) DC.	Luz.cam	1.000	0.571	0.756	0.002	**	Other
*Carum carvi* L.	Caru.car	1.000	0.429	0.655	0.026	*	Other
*Leucanthemum coronopifolium* Vill. (*s.l.*)	Leu.cor	1.000	0.429	0.655	0.013	*	Other
*Rosa pendulina* L.	Ros.pen	1.000	0.429	0.655	0.018	*	Other
*Vaccinium myrtillus* L.	Vac.myr	0.714	0.571	0.639	0.022	*	Other
**Festuco italicae-Geranietum argentei (Central Apennines)**							
*Doronicum columnae* Ten.	Dor.col	1.000	0.750	0.866	0.002	**	*Thlaspietea rotundifolii*
*Sabulina verna* (L.) Rchb. subsp. *verna*	Sab.ver	0.778	1.000	0.882	0.001	***	*Carici rupestris-Kobresietea bellardii*
*Sedum atratum* L.	Sed.atr	0.875	1.000	0.935	0.001	***	*Carici rupestris-Kobresietea bellardii*
*Potentilla crantzii* (Crantz) Beck *ex* Fritsch subsp. *crantzii*	Pot.cra	0.435	1.000	0.660	0.011	*	*Carici rupestris-Kobresietea bellardii*
*Achillea barrelieri * (Ten.) Sch. Bip. subsp. *barrelieri*	Ach.bar	1.000	0.750	0.866	0.001	***	*Leontopodio nivalis-Elynion myosuroidis*
*Erigeron epiroticus* (Vierh.) Halácsy	Eri.epi	1.000	0.750	0.866	0.001	***	*Leontopodio nivalis-Elynion myosuroidis*
*Silene acaulis* (L.) Jacq. subsp. *bryoides* (Jord.) Nyman	Sil.aca.bri	0.840	1.000	0.917	0.001	***	*Leontopodio nivalis-Elynion myosuroidis*
*Leontopodium nivale* (Ten.) Hand.-Mazz.	Leo.niv	1.000	0.500	0.707	0.007	**	*Leontopodio nivalis-Elynion myosuroidis*
*Omalotheca diminuta* (Braun-Blanq.) Bartolucci *et* Galasso	Oma.dim	1.000	0.500	0.707	0.005	**	*Leontopodio nivalis-Elynion myosuroidis*
*Carex kitaibeliana* Degen *ex* Bech.	Car.kit	1.000	1.000	1.000	0.001	***	*Festuco-Seslerietea*
*Edraianthus graminifolius* (L.) A. DC. subsp. *graminifolius*	Edr.gra	1.000	0.750	0.866	0.001	***	*Festuco-Seslerietea*
*Ranunculus breyninus* Crantz	Ran.bre	1.000	1.000	1.000	0.001	***	*Festuco-Seslerietea*
*Alchemilla nitida* Buser	Alc.nit	1.000	0.500	0.707	0.007	**	*Festuco-Seslerietea*
*Draba aizoides* L. subsp. *aizoides*	Dra.aiz	0.840	0.750	0.794	0.003	**	*Festuco-Seslerietea*
*Pulsatilla alpina* (L.) Delarbre subsp. *millefoliata* (Bertol.) D.M. Moser	Pul.alp	1.000	0.500	0.707	0.010	**	*Festuco-Seslerietea*
*Festuca violacea *Ser. ex Gaudin subsp. *italica* Foggi, Gr. Rossi et Signorini	Fes.vio	1.000	1.000	1.000	0.001	***	*Festuco-Seslerietea*
*Plantago atrata* Hoppe subsp. *atrata*	Pla.atr	1.000	1.000	1.000	0.001	***	D *Nardetea strictae*
*Taraxacum apenninum* (Ten.) DC.	Tar.ape	1.000	0.750	0.866	0.001	***	*Nardetea strictae*
*Crepis aurea* (L.) Cass. subsp. *glabrescens* (Caruel) Arcang.	Cre.aur	0.710	0.750	0.730	0.003	**	*Nardetea strictae*
*Campanula tanfanii* Podlech	Cam.tan	1.000	0.500	0.707	0.010	**	*Asplenietea trichomanis*
*Asplenium viride* Huds.	Asp.vir	0.778	0.500	0.624	0.035	*	*Asplenietea trichomanis*
*Globularia meridionalis* (Podp.) O. Schwarz	Glo.mer	1.000	0.750	0.866	0.001	***	*Festuco-Brometea*
*Helictochloa praetutiana* (Parl. *ex* Arcang.) Bartolucci, F. Conti, Peruzzi *et* Banfi subsp. *praetutiana*	Hel.pra	1.000	0.750	0.866	0.001	***	*Festuco-Brometea*
*Koeleria australis* A. Kern	Koe.aus	1.000	0.750	0.866	0.001	***	*Festuco-Brometea*
*Armeria gracilis* Ten. subsp. *gracilis*	Arm.gra	0.814	1.000	0.902	0.001	***	Other
*Gentianella columnae * (Ten.) Holub	Gen.col	1.000	1.000	1.000	0.001	***	Other
*Poa molinerii* Balb.	Poa.mol	1.000	1.000	1.000	0.001	***	Other
*Potentilla brauneana * Hoppe	Pot.bra	1.000	0.750	0.866	0.001	***	Other
*Saxifraga adscendens* L. subsp. *adscendens*	Sax.ads	1.000	1.000	1.000	0.001	***	Other
*Cystopteris fragilis* (L.) Bernh.	Cys.fra	0.923	0.750	0.832	0.002	**	Other
*Anthyllis vulneraria* L. subsp. *nana* (Ten.) Tammaro	Ant.vul.nan	1.000	0.500	0.707	0.011	*	Other

Original data source: *Salici retusae-Geranietum argentei* Surina 2005 from Table 11: rels. 1–16 in Surina (2005), Krn Mountain (Julian Alps) in northwestern Slovenia; *Sesleriello sphaerocephalae-Geranietum argentei* Sutter ex Ballelli, Tesei, Pennesi et Allegrezza ass. nova from Table II: rels. 1–3 in Sutter (1969) [18], Mt. Serva Dolomites Alps, and from Table 11: rels. 1, 10, 11, 12 in Pignatti and Pignatti (2016) [10], Mt. Serva, Mt. Cavallo, Mt. Lastè, Dolomites Alps; *Geranio argentei-Caricetum rupestris* Tomaselli et al., 2019 from Table 4: rels. 1–5 in Tomaselli et al. (2019) [19], Mt. Cimone, Tuscan-Emilian Apennines (Northern Apennines); *Geranium argenteum* group: 7 rels. in Cortopassi thesis (2007) [11] from Northern Apennines, of which 6 rels. from Tuscan-Emilian Apennines (Mt. Vecchio; Mt. Corno alle Scale; Mt. Spogolino) are on siliciclastic substrate and the one rel. from Apuan Alps (Pania della Croce) is on calcareous substrate; *Festuco italicae-Geranietum argentei* ass. nova from Table 1: rels. 1–4 in this paper (study area, Central Apennines).

### 3.3. Validations of Syntaxa

*Sesleriello sphaerocephalae-Geranietum argentei* Sutter *ex* Ballelli, Tesei, Pennesi *et* Allegrezza *ass. nova* (*typus* rel. 1, Table II, p. 356, in Sutter 1969)

Valitaded name: “Sesleria sphaerocephala-Geranium argenteum-Ass. prov.” in Sutter (1969, p. 356) [18] (Art. 3b).

Synonym: *Seslerio sphaerocephalae*-*Geranietum argentei* Sutter 1969 (nom. inv. Art. 3b); *Seslerio caeruleae*-*Geranietum argentei* Sutter 1969 (*nom. inv.* Art. 3b).

Diagnostic taxa of the association are reported in Table II, p. 356 in Sutter (1969 [18]). They are: *Geranium argenteum* L., *Carex capillaris* L., *Carex myosuroides* Vill., *Dianthus sternbergii* Sieber ex Capelli, *Sesleriella sphaerocephala* (Ard.) Deyl (*Sesleria sphaerocephala* Ard.), and *Oxytropis carinthica* Fischer.

### 3.4. General Syntaxonomic Scheme for Alpine–Apennines Communities Considered for the Comparison

*CARICI RUPESTRIS-KOBRESIETEA BELLARDII* Ohba 1974

+*Oxytropido*-*Elynetalia* Albrecht 1969

*Oxytropido-*Elynion myosuroidis* Br.-Bl. 1950

*Sesleriello sphaerocephalae*-*Geranietum argentei* Sutter *ex* Ballelli, Tesei, Pennesi et Allegrezza ass. nova

*Geranio argentei*-*Caricetum rupestris* Tomaselli, Carbognani, Foggi, Petraglia, Rossi, Lombardi et Gennai 2019

*Leontopodio nivalis-*Elynion myosuroidis* (Blasi *et* Di Pietro in Blasi, Di Pietro, Fortini *et* Catonica 2003) Di Pietro et Mucina in Chytry, Danlël, Di Pietro, Koroleva et Mucina 2015

*Festuco italicae*-*Geranietum argentei ass. nov.*

*THLASPIETEA ROTUNDIFOLII* Br.-Bl. 1948

+*Arabidetalia coeruleae* Rübel *ex* Nordhagen 1936

*Arabidion caeruleae Br.-Bl. in Br.-Bl. *et* Jenny 1926

(=*Soldanello alpinae*-*Salicion retusae* Englisch 1999 (*syntax. syn.*)

*Salici retusae*-*Geranietum argentei* Surina 2005

*FESTUCO*-*SESLERIETEA* Barbéro-Bonin 1969

+*Seslerietalia tenuifoliae* Horvat 1930

*Geranium argenteum* group

### 3.5. Climate Change and Plant Community Conservation Notes

Climate change has a high negative impact on high-altitude habitats, especially in the Mediterranean area where the few isolated mountain peaks host plant associations characterized by endemic and rare species (e.g., Pauli et al., 2003) [48] with a narrow and fragmented areal [49]. The potential impact of climate change on the fragile and narrowly restricted *G. argenteum* plant association in the study area could alter its floristic composition and lead to its extinction. As observed in the short and long term (e.g., Frate et al., 2018) [50], climatic warming has determined the reduction in typically cryophilous species and the upward shift of thermophilous species from the lower vegetation belt (e.g., Korner 2021) [51], a process described as thermophilization (e.g., Stanisci et al., 2011) [52]. Recently, the decline of the alpine cushion plant *Silene acaulis* at its southern limit of distribution in the Apennines was documented by Bonanomi et al., 2023 [53] as being strongly linked to particularly intense heatwaves. The vulnerability of high-altitude species such as *G. argenteum* is also amplified by the high fragmentation of the species at its southern limit of distribution and the absence of specific dispersal mechanisms (mean dispersal distance <1 m) [54]. Dispersal distance can play a key role in the persistence and survival of rare species confined to particular microhabitats, as demonstrated by seven endangered and vulnerable taxa in the central Apennines [49]. In order to conserve the microhabitats that host the *G. argenteum* plant community in the study area, it will be essential to monitor over time any changes in the floristic composition of the community and the population size of *G. argenteum*. In particular for *G. argenteum*, it is also important to collect seeds for ex situ conservation for possible population reinforcement.

## Figures and Tables

**Table 2:** Topographical, biological, and distributive parameters of the communities characterized by *Geranium argenteum.* Higher values are indicated in bold. Abbreviations: Sal-Ger. (*Salici retusae-Geranietum argentei*); Sesl-Ger. (*Sesleriello sphaerocephalae-Geranietum argentei*); Ger. gr. (*Geranium argenteum* group); Ger-Car. (*Geranio argentei-Caricetum rupestris*); Fest-Ger. (*Festuco italicae-Geranietum argentei*, study area).

Geography	Alps		Apennines		
	Julian	Dolomites	Northern	Northern	Central
Plant communities	Sal-Ger.	Sesl-Ger.	Ger-Car.	Ger. gr.	Fest-Ger.
No. of relevés	16	7	5	7	4
**Mean Elevation** (m. a.s.l.)	1806	**2105**	1988	1818	**2058**
**Mean Slope** (°)	16.3	**51.7**	12.0	39.0	35.0
**Substrate** (C: calcareous; S: siliciclastic)	C	C	S	S, C	C
**Mean Species Cover** (%)	87.5	**93.3**	90.0	42.1	60.0
**Mean richness**	17.0	29.4	10.0	**38.0**	34.0
***Geranium argenteum* mean cover** (%)	38.0	15.0	4.2	7.3	**44.4**
**Chorological type mean richness** (%)					
Endemic	14.5	19.8	21.1	19.5	**30.0**
Boreal	**31.6**	26.4	26.3	17.9	13.3
South-European orophytes	32.9	36.3	36.8	34.1	**38.3**
European orophytes	**11.8**	8.8	5.3	1.6	3.3
Mediterranean	1.3	4.4	5.3	5.7	**8.3**
Eurasian	5.3	4.4	5.3	**16.3**	3.3
Cosmopolitan	2.6	0.0	0.0	**4.9**	3.3

## Data Availability

All data generated or analyzed during this study are included in this published article and its supplementary information files.

## Supplementary Materials

The following supporting information can be downloaded at: https://www.mdpi.com/article/10.3390/life13122273/s1, Figure S1: Dendrogram obtained from the classification of the relevés groups of the Central Apennines communities, from the high mountain to alpine belts; Table S1: Synoptic table of the plant communities occurring in the alpine and subalpine belts of the Central Apennines; Table S2: Chorological spectrum concerning the species of the new plant association *Festuco italicae-Geranietum argentei*; Table S3: Weight of species and the relative % of variance explained by each one.

## References

[1] Aeschimann D., Lauber K., Moser D.M., Theurillat J.P. (2004). Flora Alpina. Volumes 1–3.

[2] Pignatti S., Guarino R., La Rosa M. (2017). Flora d’Italia, 2nd Edition.

[3] Dengler J., Jansen F., Chusova O., Hüllbusch E., Nobis M.P., Van Meerbeek K., Axmanová I., Bruun H.H., Chytrý M., Guarino R. (2023). Ecological Indicator Values for Europe (EIVE) 1.0. Veg. Classif. Surv..

[4] Pignatti S. (2005). Valori di bioindicazione delle piante vascolari della flora d’Italia. Braun-Blanquetia.

[5] Chiarugi A. (1937). Sul limite boreale dell’area geografica del Geranium argenteum L. nelle Dolomiti Occidentali. N. Giorn. Bot. Ital..

[6] Ferrarini E. (1967). Studi sulla vegetazione di altitudine delle Alpi Apuane (Seconda parte: Le Apuane sud-orientali). Webbia.

[7] Ansaldi M., Cortopassi L., Garbari F. (2008). Ecologia della conservazione di popolamenti apuano-appenninici di *Geranium argenteum* L.. Atti Soc. Tosc. Sci. Nat. Mem..

[8] Tomaselli M., Ross I.G., Dowgiallo G. (2000). Phytosociology and ecology of the Festuca puccinellii-grasslands in the northern Apennines (N-Italy). Bot. Helv..

[9] Gams H. (1933). Der tertiäre Grundstock der Alpenflora. Der Begriff des alpigenen Florenelements. Jahrb Verein Schutz Alpenpflanz Tiere..

[10] Pignatti E., Pignatti S. (2014). Plant Life of the Dolomites. Vegetation Structure and Ecology. Publication of the Museum of Nature South Tyrol 8.

[11] Cortopassi L. (2007). Variabilità infraspecifica in popolamenti apuano-appenninici di *Geranium argenteum* L.. Master’s Thesis.

[12] Alonzi A., Ercole S., Piccini C. La protezione delle specie della flora e della fauna selvatica: Quadro di riferimento legislativo regionale. APAT. 2006, Rapporti 75/2006. https://www.researchgate.net/publication/233951463_La_protezione_delle_specie_della_flora_e_della_fauna_selvatica_quadro_di_riferimento_legislativo_regionale.

[13] Bartolucci F., Peruzzi L., Galasso G., Albano A., Alessandrini A., Ardenghi N.M.G., Astuti G., Bacchetta G., Ballelli S., Banfi E. (2018). An updated checklist of the vascular flora native to Italy. Plant Biosyst..

[14] Bertoloni A. (1847). *Flora* *italica*. Bononiae.

[15] Dell’Orso M., Rossetti A., Tescarollo P. (2010). Il Giardino Della Sibilla. Guida ai fiori del Parco Nazionale dei Monti Sibillini. Collana Le Gemme. Guide Alla Natura Delle Regioni d’Italia.

[16] Ballelli S., Cesaretti S. Aggiornamento dell’elenco floristico di supporto alle aree floristiche della Regione Marche. B.U.R. Marche, Anno XLV, 2014, n. 42 All. C: 8569–8586.

[17] Surina B. (2005). Subalpinska in alpinska vegetacija Krnskega pogorja v Julijskih Alpah. Scopolia.

[18] Sutter R. (1969). Ein Beitrag zur Kenntnis der soziologischen Bindung süd-südostalpiner Reliktendemismen. Acta Bot. Croat..

[19] Tomaselli M., Carbognani M., Foggi B., Petraglia A., Rossi G., Lombardi L., Gennai M. (2019). The primary grasslands of the northern Apennine summits (N-Italy): A phytosociological and ecological survey. Tuexenia.

[20] Dakskobler I. (2011). Novosti v flori zahodne Slovenije (Primorska). Hladnikia.

[21] Surina B. (2005). Phytogeography and syntaxonomy of snow-bed vegetation on calcareous soils in the South-eastern Alps: A numerical approach. Ann. Ser. Hist. Nat..

[22] Pignatti E., Pignatti S. (2016). Plant Life of the Dolomites: Vegetation Tables.

[23] eVeg database. 2016–2023. Release 2.1.0. Clermont-Ferrand (FRANCE). http://www.e-veg.net/app/12250.

[24] Pierantoni P.P., Deiana G., Galdenzi S. (2013). Geological map of the Sibillini Mountains (Umbria-Marche Apennines, Italy). Ital. J. Geosci..

[25] Rivas-Martínez S., Sáenz S.R., Penas À. (2011). Worldwide bioclimatic classification system. Glob. Geobot..

[26] Pesaresi S., Biondi E., Casavecchia S. (2017). Bioclimates of Italy. J. Maps.

[27] Braun-Blanquet J. (1932). Plant Sociology: The Study of Plant Communities.

[28] Theurillat J.-P., Willner W., Fernández-González F., Bültmann H., Čarni A., Gigante D., Mucina L., Weber H. (2021). International Code of Phytosociological Nomenclature. 4th edition. Appl. Veg. Sci..

[29] Biondi E., Blasi C., Allegrezza M., Anzellotti I., Azzella M.M., Carli E., Casavecchia S., Copiz R., Delvico E., Facioni L. (2014). Plant communities of Italy: The Vegetation Prodrome. Plant Biosyst..

[30] Biondi E., Allegrezza M., Casavecchia S., Galdenzi D., Gasparri R., Pesaresi S., Poldini L., Sburlino G., Vagge I., Venanzoni R. (2015). New syntaxonomic contribution to the Vegetation Prodrome of Italy. Plant Biosyst..

[31] Chytrý M., Daniëls F.J.A., Di Pietro R., Koroleva N., Mucina L. (2015). Nomenclature adjustments and new syntaxa of the Arctic, alpine and oro-Mediterranean vegetation. Hacquetia.

[32] Mucina L., Bultmann H., Dierßen K., Theurillat J.P., Raus T., Carni A., Sumberova K., Willner W., Dengler J., Garcıa R.G. (2016). Vegetation of Europe: Hierarchical floristic classification system of vascular plant, bryophyte, lichen, and algal communities. App. Vegl. Sci..

[33] van der Maarel E. (1979). Transformation of cover-abundance values in phytosociology and its effects on community similarity. Vegetatio.

[34] Oksanen J., Blanchet F.G., Friendly M., Kindt R., Legendre P., McGlinn D., Minchin P.R., O’Hara R.B., Simpson G.L., Solymos P. Vegan: Community Ecology Package. R Package Version 2.5-7 2020. https://CRAN.R-project.org/package=vegan.

[35] R Core Team (2021). R: A Language and Environment for Statistical Computing.

[36] De Cáceres M., Legendre P. (2009). Associations between species and groups of sites: Indices and statistical inference. Ecology.

[37] Dufrêne M., Legendre P. (1997). Species assemblages and indicator species: The need for a flexible asymmetrical approach. Ecol. Monogr..

[38] Conti F. (1992). Alcune piante di particolare interesse fitogeografico rinvenute sulle Mainarde (Lazio e Molise). L’uomo e l’ambiente Camerino.

[39] Blasi C., Di Pietro R., Fortini P., Catonica C. (2003). The main plant community types of the alpine belt of the Apennine chain. Plant Biosyst..

[40] Lancioni A., Facchi J., Taffetani F. (2011). Syntaxonomical analysis of the Kobresio oricumdes-Seslerietea caeruleae and Carici rupestris-Kobresietea bellardii classes in the central southern Apennines. Fitosociologia.

[41] Biondi E., Ballelli S., Allegrezza M., Taffetani F., Frattaroli A.R., Guitan J., Zuccarello V. (1999). La vegetazione di Campo Imperatore (Gran Sasso d’Italia). Braun-Blanquetia.

[42] Biondi E., Ballelli S., Allegrezza M., Taffetani F. (2000). La vegetazione del Corno Grande nel Gran Sasso d’Italia (Appennino centrale). Fitosociologia.

[43] Biondi E., Allegrezza M., Taffetani F., Ballelli S., Zuccarello V. (2002). Excursion to the National Park of Gran Sasso and Monti della Laga. Fitosociologia.

[44] Blasi C., Di Pietro R., Pelino G. (2005). The vegetation and landscape of alpine belt karst-tectonic basin in the Majella mountain (central Apennines). Plant Biosyst..

[45] Di Pietro R., Pelino G., Stanisci A., Blasi C. (2008). Phytosociological features of *Adonis distorta* and *Trifolium oricum* subsp. praetutianum, two endemics of the Apennines (Peninsular Italy). Acta Bot. Croat..

[46] Catorci A., Ballelli S., Gatti R., Vitanzi A. (2008). Studio fitosociologico delle praterie della Valle dell’Ambro (Parco Nazionale dei Monti Sibillini, Italia Centrale). Inform Bot. Ital..

[47] Costanzo E., Furnari F., Tomaselli V. (2009). A phytosociological survey on the main plant community types of alpine and sub-alpine belt in the Sibillini Mountains (Central Apennines, Italy). Lazaroa.

[48] Pauli H., Gottfried M., Grabherr G. (2014). Effects of climate change on the alpine and nival vegetation of the Alps. J. Mt. Ecol..

[49] Di Musciano M., Di Cecco V., Bartolucci F., Conti F., Frattaroli A.R., Di Martino L. (2020). Dispersal ability of threatened species affects future distributions. Plant Ecol..

[50] Frate L., Carranza M.L., Evangelista A., Stinca A., Schaminée J.H.J., Stanisci A. (2018). Climate and land use change impacts on Mediterranean high-mountain vegetation in the Apennines since the 1950s. Plant Ecol. Divers..

[51] Körner C. (2021). Alpine Plant Life: Functional Plant Ecology of High Mountain Ecosystems; with 47 Tables.

[52] Stanisci A., Carranza M.L., Pelino G., Chiarucci A. (2011). Assessing the diversity pattern of cryophilous plant species in high elevation habitats. Plant Ecol..

[53] Bonanomi G., Idbella M., Allegrezza M., Tesei G. (2023). Dieback of the cushion plant Silene acaulis at its southern limit of distribution in the Apennines. Alp. Bot..

[54] Lososová Z., Axmanová I., Chytrý M., Midolo G., Abdulhak S., Karger D.N., Renaud J., Van Es J., Vittoz P., Thuiller W. (2023). Seed dispersal distance classes and dispersal modes for the European flora. Glob. Ecol. Biogeogr..

