# Supplementary material for: Floristic–Vegetational Features of Geranium argenteum, an Alpine–Apennine Species at Its Limit of Distribution in the Apennines
Source: Life (Basel). 2023 Nov 28;13(12):2273. doi: 10.3390/life13122273 (PMC10744573; doi:10.3390/life13122273)
Supplement: Supplementary file 1 [file life-13-02273-s001.zip › life-2693359-supplementary.pdf]

Supplementary Materials

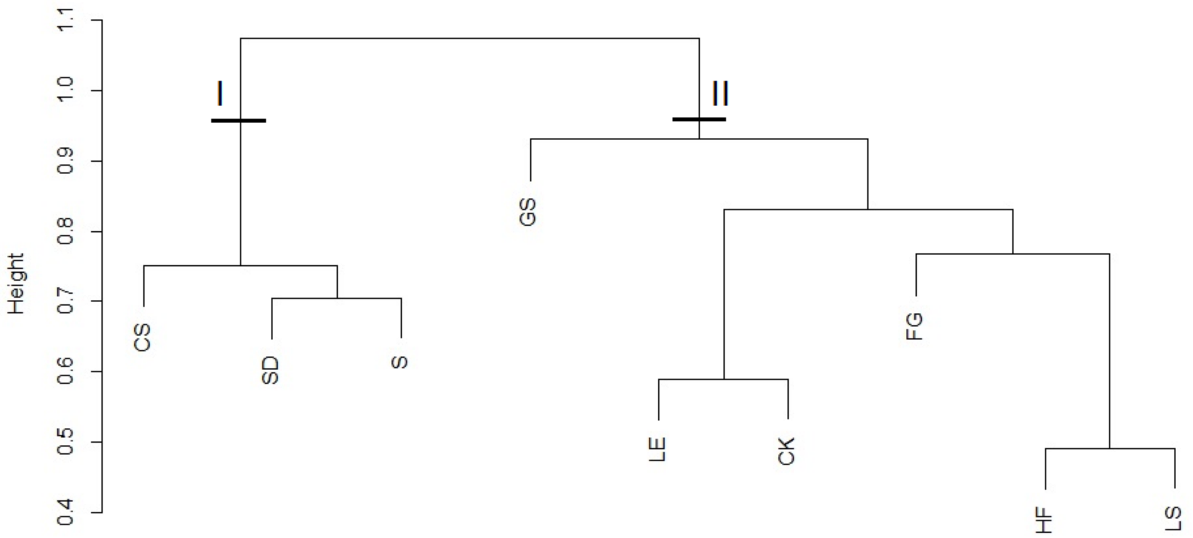

Figure S1. Dendrogram obtained from the classification of the relevés groups of the Central Apennines communities, from the high mountain to alpine belts, belonging to the *Carici rupestris-Kobresietea bellardii* and *Festuco-Seslerierea* classes. Cluster I: *Carici rupestris-Kobresietea bellardii* class; Cluster II: *Festuco-Seslerietea* class. Legend of plant associations: (LE) *Leontopodio nivalis-Elynetum myosuroidis* (typus of the *Leontopodio nivalis-Elynion myosuroidis* alliance); (GS) *Galio majellensis-Silenetum acaulis;* (CK) *Caricetum kitaibelianae-rupestris;* (FG) *Festuco italicae-Geranietum argentei* (ass. nova, study area); (LS) *Leontopodio nivalis-Seslerietum juncifoliae*; (HF) *Helianthemo alpestris-Festucetum italicae*; (SD) *Seslerio apenninae-Dryadetum octopetalae;* (S) *Seslerietum apenninae* (typus of the *Seslerion apenninae* alliance); (CS) *Carici humilis-Seslerietum apenninae* (typus of the *Carici humilis-Seslerion apenninae* alliance).

Table S1. Synoptic table with frequence values in percent of the plant communities occurring in the alpine and subalpine belts of the central Apennines. Column 1 - *Festuco italicae-Geranietum argentei* ass. nova from Table 1: rels. 1-4 in this paper (study area, Central Apennines); column 2 - *Galio magellensis-Silenetum acaulis* Blasi et al. 2003 from Table 5: rels. 1-8 in Blasi et al. (2003), Gran Sasso mountains (Central Apennines); column 3 - *Helianthemo alpestris-Festucetum italicae* Blasi et al. 2005 from Table VII: rels. 1-13 in Blasi et al. (2005), Majella mountans (Central Apennines); column 4 - *Leontopodio nivalis-Seslerietum juncifoliae* Blasi et al. 2005 from Table VI: rels.. 1-13 in Blasi et al. (2005), Majella mountains (Central Apennines); column 5 - *Seslerio apenninae-Dryadetum octopetalae* Biondi et al. 1999 from Tab. 5: rels. 1-13 in Biondi et al. (1999), Gran Sasso mountains (Central Apennines); column 6 - *Leontopodio nivalis-Elynetum myosuroidis* Feoli Chiapella et Feoli 1977 (column 6) from Table 1: rels. 1-8 in Biondi et al. (2000), Gran Sasso mountains (Central Apennines); column 7 - *Caricetum kitaibelianae-rupestris* Biondi et al. 2000 from Table 2: rels. 1-12 in Biondi et al. (2000), Gran Sasso (Central Apennines).

No. of column	1	2	3	4	5	6	7
No. relevés	4	8	13	13	13	8	12
Festuco italicae-Geranietum argentei *ass. nova*							
Festuca violacea Ser. *ex* Gaudin subsp. italica Foggi, Gr. Rossi *et* Signorini	100	25	100	77		75	83
Achillea barrelieri (Ten.) Sch. Bip. subsp. barrelieri	75	87	15		15		33
Gentianella columnae (Ten.) Holub	100			8			
Geranium argenteum L.	100						
Potentilla brauneana Hoppe	75						
Galio magellensis-Silenetum acaulis Blasi *et al.* 2003							
Galium magellense Ten.	25	87		8			33
Androsace vitaliana (L.) Lapeyr. subsp. praetutiana (Buser *ex* Sünd) Kress		100				12	33
Saxifraga aizoides L.		50				12	33
Festuca alfrediana Foggi *et* Signorini subsp. ferrariniana Foggi, Parolo *et* Gr. Rossi		75					
Gentiana utriculosa L.		37					
Cerastium thomasii Ten.		100					
Heliantemo alpestris-Festucetum italicae Blasi *et al.* 2005							
Helianthemum oelandicum (L.) Dum.Cours. subsp. alpestre (Jacq.) Ces.			92	85	23	87	83
Sempervivum arachnoideum L.			85	85		12	33
Poa molineri Balb.	100		61	61			
Trifolium thalii Vill.			23			12	
Leucanthemum trydactylites (A. Kern. *et* Huter *ex* Porta *et* Rigo) Huter, Porta *et* Rigo			61				
Trifolium prantense L. subsp. semipurpureum (Strobl) Pignatti			23				
Leontopodio nivalis-Seslerietum juncifoliae Blasi *et al.* 2005							
Sesleria juncifolia Suffren subsp. juncifolia	50		54	100	100	50	92
Aster alpinus L. subsp. alpinus	25		31	85		50	42
Ranunculus breyninus Crantz	100		23	85			17
Iberis saxatilis L. subsp. saxatilis			23	92	15		
Carex humilis Leyss.				77	31		
Seslerio apenninae-Dryadetum octopetalae Biondi *et al.* 1999							
Dryas octopetala L. subsp. octopetala				15	100		33
Carex mucronata All.					61		
Gentiana dinarica Beck					69		
Lomelosia graminifolia (L.) Greuter *et* Burdet subsp. graminifolia					61		
Ranunculus thora L.					31		
Leontopodio nivalis-Elynetum myosuroidis Feoli Chiapella *et* Feoli 1977							
Carex myosuroides Vill.		5		15		100	17

Antennaria dioica (L.) Gaetn.						50	8
Armeria gracilis Ten. subsp. majellensis (Boiss.) Arrigoni						50	42
Alchemilla colorata Buser						75	
Caricetum kitaibelianae-rupestris Biondi *et al*. 2000							
Carex rupestris All.					8	50	92
Potentilla apennina Ten. subsp. apennina				8	15		42
Artemisia eriantha Ten.							42
Gentiana orbicularis Schur							33
Leontopodio-Elynion, Oxytropido-Elynetalia, Carici rupestris-Kobresietea bellardii							
Silene acaulis (L.) Jacq. subsp. bryoides (Jord.) Nyman	100	100	77	69	8	87	92
Erigeron epiroticus (Vierh.) Halácsy	75	25	61	46		62	42
Carex kitaibeliana Degen *ex* Bech.	100		100	85	85	100	92
Sabulina verna (L.) Rchb. subsp. verna	100		92	69	31	50	42
Sedum atratum L.	100	25	31			37	58
Leontopodium nivale (Ten.) É. Huet *et* A. Huet *ex* Hand.-Mazz.	50		100	100	31	12	
Potentilla crantzii (Crantz) Beck *ex* Fritsch subsp. crantzii	100		61	38		75	17
Bistorta vivipara (L.) Delarbre			8	8	8	100	33
Omalotheca diminuta (Braun-Blanq.) Bartolucci *et* Galasso	50	5	61				
Pedicularis verticillata L. subsp. verticillata		5				25	33
Oxytropis campestris (L.) DC.			77	85			
Trifolium noricum Wulfen subsp. praetutianum (Guss. *ex* Savi) Arcang.						12	8
Carex ericetorum Pollich						62	33
Oxytropis neglecta J. Gay *ex* Ten.			8				
Gentiana nivalis L.						25	
Saxifraga exarata Vill. subsp. ampullacea (Ten.) D.A. Webb							17
Festuco-Seslerietea							
Draba aizoides L. subsp. aizoides	75	62	85	85	15	100	75
Edraianthus graminifolius (L.) DC. *ex* Meisn. subsp. graminifolius	75	25	61	92	77	75	100
Androsace villosa L. subsp. villosa	50		85	54	85	25	33
Pulsatilla alpina (L.) Delarbre subsp. millefoliata (Bertol.) D.M. Moser	50		85	31	15	12	33
Trinia dalechampii (Ten.) Janch.	25		83	85	8	62	50
Gentiana verna L. subsp. verna	50		46	31		87	33
Pedicularis elegans Ten.	25			92	8	37	17
Euphrasia salisburgensis Funck *ex* Hoppe	75		8	23		12	
Phyteuma orbiculare L.			15		8	25	8
Polygala alpestris Rchb. subsp. alpestris			8	8			
Galium anisophyllum Vill.						37	8
Saxifraga caesia L.					23		17
Coeloglossum viride (L.) Hartm.						12	17
Thesium parnassi A. DC.				8	23		
Alchemilla nitida Buser	50						
Paronychia kapela (Hacq.) A. Kern. subsp. kapela	25						
Carduus defloratus L. subsp. carlinifolius (Lam.) Ces.	25						
Bellidiastrum michelii Cass.	75						
Helianthemum nummularium (L.) Mill. subsp. grandiflorum (Scop.) Schinz *et* Thell.				8			
Biscutella laevigata L. subsp. laevigata							8
Carex firma Host					8		
Nardetea strictae and ***Juncetea trifidi***							
Plantago atrata Hoppe subsp. atrata	100		15	8		37	

Luzula italica Parl.	25		8			75	8
Campanula scheuchzeri Vill. subsp. scheuchzeri			31	8			17
Pilosella lactucella (Wallr.) P.D. Sell *et* C .West subsp. nana (Scheele) M. Laínz	25					37	
Botrychium lunaria (L.) Sw.	25					25	
Anthoxanthum nipponicum Honda						25	8
Taraxacum apenninum (Ten.) DC.	75						
Crepis aurea (L.) Cass. subsp. glabrescens (Caruel) Arcang.	75						
Asplenietea trichomanis							
Saxifraga paniculata Mill.	75	5	69	85	8	12	33
Saxifraga oppositifolia L. subsp. oppositifolia		5	8	23	8		8
Campanula tanfanii Podlech	50				8		
Asplenium viride Huds.	50						
Cystopteris fragilis (L.) Bernh.	75						
Primula auricula L.							33
Thlaspietea rotundifolii							
Saxifraga adscendens L. subsp. adscendens	100		8	31	8	12	8
Ranunculus brevifolius Ten.	25	25	23				
Carum heldreichii Boiss.					38	12	8
Valeriana saliunca All.			8	15			
Robertia taraxacoides (Loisel.) DC.	25	25					
Noccaea stylosa (Ten.) Rchb.		37	8				
Scorzoneroides montana (Lam.) Holub			8		8		
Leucopoa dimorpha (Guss.) H. Scholz *et* Foggi	25						
Doronicum columnae Ten.	75						
Arabis alpina L. subsp. caucasica (Willd.) Briq.	25						
**other species**							
Anthyllis montana L. subsp. jacquinii (Rchb. *f.*) Rohlena	25			15	69	12	33
Thymus praecox Opiz subsp. polytrichus (A. Kern *ex* Borbás) Jalas	75	50	92	46	8		33
Myosotis graui Selvi	25	62	23	23		62	17
Poa alpina L. subsp. alpina		87	46	54	8	87	58
Salix retusa L.			15	23	8	12	17
Cerastium arvense L. subsp. suffruticosum (L.) Ces.	50		69	61			8
Viola eugeniae Parl. subsp. eugeniae			31	15		37	8
Helictochloa praetutiana (Parl. *ex* Arcang.) Bartolucci, F. Conti, Peruzzi *et* Banfi subsp. praetutiana	75		83	69	46		
Anthyllis vulneraria L. subsp. nana (Ten.) Tammaro	50				8	50	58
Cynanchica pyrenaica (L.) P. Caputo *et* Del Guacchio subsp. neglecta (L.) P. Caputo *et* Del Guacchio	25				8	12	
Globularia meridionalis (Podp.) O. Schwarz	75				61		8
Oreojuncus monanthos (Jacq.) Záv. Drábk. *et* Kirschner	50			23		37	
Armeria canescens (Host) Ebel		37	69	46			
Pilosella officinarum Vaill.			31	8	8		
Dianthus sylvestris Wulfen subsp. sylvestris					8	12	33
Helianthemum oelandicum (L.) Dum. Cours. subsp. incanum (Willk.) G. López	25				61		
Koeleria australis A. Kern	75				8		
Anthyllis vulneraria L. subsp. pulchella (Vis.) Bornm.			100	92			
Arenaria grandiflora L. subsp. grandiflora			8	31			
Pinus mugo Turra subsp. mugo			8	8			
Cerastium arvense L. subsp. strictum (W.D.J. Koch) Gremli					8		33
Juniperus communis L.					15	12	
Armeria gracilis Ten. subsp. gracilis	100						

Carlina acaulis L. subsp. caulescens (Lam.) Schübl. *et* G. Martens	25				
Ziziphora granatensis (Boiss. *et* Reut.) Melnikov subsp. alpina (L.) Bräuchler *et* Gutermann	25				
Anthemis cretica L. subsp. columnae (Ten.) Franzén				37	
Festuca laevigata Gaudin		8			
Veronica aphylla L. subsp. aphylla					33
Astrantia pauciflora Bertol. subsp. tenorei (Mariotti) Bechi *et* Garbari				25	
Arctostaphylos uva-ursi (L.) Spreng.			15		
Linum capitatum Kit. *ex* Schult. subsp. serrulatum (Bertol.) Hartvig			15		
Carex macrolepis DC.			23		
Leontodon hispidus L.			31		
Brachypodium genuense (DC.) Roem. *et* Schult.			23		
Anthyllis vulneraria L. subsp. weldeniana (Rchb.) Cullen			23		
Teucrium montanum L.			23		
Linum catharticum L. subsp. catharticum			23		
Coronilla minima L.			15		
Bromopsis erecta (Huds.) Fourr.			15		
Hieracium bifidum Kit. ex Hornem.			8		
Lotus corniculatus L.			8		

Table S2. Chorological spectrum of the species in Table 1. Legend: (n) percentage of species; (f) percentage frequency; (c) species cover percentage

**Chorological type**	**n %**	**f %**	**c %**
Endemic Italian and subendemic	25.0	25.2	71.3
Boreal	15.0	16.8	11.4
South European orophytes	38.3	38.5	13.6
European Orophytes	5.0	6.3	1.2
Mediterranean	11.7	9.8	1.8
Eurasian	1.7	0.7	0.1
Cosmopolitan	3.3	2.7	0.5

Table S3. Weight of species shown in the PCA of Figure 5 with respect to the three main components identified (axes PC1, PC2 and PC3) and the relative % of variance explained by each one. The full name of the abbreviated species are reported in Table 4

	Species weight PC1 (%)	Variance explain by species PC1 (%)	Species weight PC2 (%)	Variance explain by species PC2 (%)	Species weight PC3 (%)	Variance explain by species PC3 (%)
Sal.retu	10.08	1.84	0.11	0.01	4.86	0.49
Bis.viv	9.80	1.79	0.05	0.01	0.01	0.00
Tri.pal	7.13	1.30	0.08	0.01	3.21	0.32
Par.pal	7.00	1.28	0.09	0.01	3.13	0.32
Car.rup	4.65	0.85	30.02	3.38	2.19	0.22
Hel.oel.inc	2.89	0.53	0.09	0.01	3.30	0.33
Sax.pan	2.76	0.50	0.36	0.04	0.61	0.06
Thy.pra	2.52	0.46	0.68	0.08	0.46	0.05
Ger.arg	2.46	0.45	0.64	0.07	0.43	0.04
Gal.ani	2.45	0.45	0.02	0.00	0.07	0.01
Gen.ani	2.43	0.44	0.11	0.01	0.53	0.05
Ast.alp	2.41	0.44	3.65	0.41	1.11	0.11
Fes.lae	2.12	0.39	4.30	0.48	1.05	0.11
Ant.nip	2.00	0.37	0.12	0.01	0.77	0.08
Eup.sal	1.68	0.31	0.07	0.01	1.26	0.13
Sol.alp	1.67	0.30	0.21	0.02	1.98	0.20
Ses.jun	1.64	0.30	2.95	0.33	0.19	0.02
Alc.tra	1.32	0.24	8.12	0.91	1.71	0.17
Ach.cla	1.28	0.23	0.01	0.00	0.01	0.00
Ped.ver	1.25	0.23	0.02	0.00	0.12	0.01
Cer.arv	1.22	0.22	2.22	0.25	0.06	0.01
Bra.gen	1.20	0.22	2.48	0.28	0.45	0.05
Pot.cra	1.16	0.21	0.09	0.01	0.87	0.09
Poa.alp	1.15	0.21	2.62	0.29	2.98	0.30
Fes.nit	1.11	0.20	0.01	0.00	0.60	0.06
Agr.rup	1.09	0.20	0.01	0.00	0.82	0.08
Ach.alp	0.92	0.17	1.90	0.21	0.35	0.04
Alc.cin	0.88	0.16	1.80	0.20	0.44	0.04
Fes.ric	0.83	0.15	2.96	0.33	0.93	0.09
Bel.mic	0.77	0.14	2.03	0.23	0.48	0.05
Fes.vio	0.33	0.06	0.25	0.03	1.39	0.14
Alc.fla	0.17	0.03	5.22	0.59	5.47	0.55
Sab.ver	0.11	0.02	0.03	0.00	1.94	0.20
Sil.aca	0.08	0.01	0.18	0.02	2.66	0.27
Dry.oct	0.06	0.01	0.19	0.02	2.00	0.20
Ses.cae	0.03	0.01	0.34	0.04	4.05	0.41
Car.myo	0.02	0.00	0.57	0.06	4.74	0.48
Ses.sph	0.02	0.00	0.25	0.03	1.96	0.20
Cha.alp	0.02	0.00	0.28	0.03	2.36	0.24
Sax.cru	0.01	0.00	0.20	0.02	2.64	0.27
Dia.ste	0.01	0.00	0.17	0.02	1.50	0.15
